# Close relationship between the genera *Sinhomidia* and *Homidia* (Collembola, Entomobryidae) revealed by adult and first instar characters, with description of a new *Sinhomidia* species

**DOI:** 10.3897/zookeys.872.29815

**Published:** 2019-08-20

**Authors:** Zhi-Xiang Pan, Chen-Chong Si, Shu-Sheng Zhang

**Affiliations:** 1 School of Life Sciences, Taizhou University, Taizhou, Zhejiang province 318000, China Taizhou University Taizhou China; 2 Taizhou Foreign Language School, Taizhou, Zhejiang 318000, China Taizhou Foreign Language School Taizhou China; 3 Wuyanling National Nature Reserve, Wenzhou, Zhejiang 325500, China Wuyanling National Nature Reserve Wenzhou China

**Keywords:** chaetotaxy, Entomobryinae, *Sinhomidia uniseta* sp. nov., taxonomy

## Abstract

A third species of the genus *Sinhomidia* is described from South China: *S. uniseta***sp. nov.** This new species can be distinguished from the two other species of the genus by the following characters: colour pattern, single labial chaeta M, chaetotaxy on terga and ventral tube, unguis with three inner teeth, and 15 clypeal ciliated chaetae. Also, the chaetotaxy of the first instar of *Sinhomidia* is described for the first time in the present paper, and confirms the close relationship between *Sinhomidia* and *Homidia*. A key to species of *Sinhomidia* is provided.

## Introduction

The genus *Sinhomidia* was defined by Zhang et al. (2009), based on the type species *Acanthocyrtus bicolor* (Yosii, 1965). This genus is characterised by 8+8 eyes; scales pointed with coarse striations; dental spines present on inner dens; clavate tenent hairs; mucro bidentate with subapical tooth larger than apical one, and basal spine reaching subapical tooth; abdominal segment II/III/IV with 2/3/2 bothriotricha, and bothriotrichal complex with slightly modified accessory microchaetae; abdominal segment IV with anterior eyebrow-like macrochaetae (Zhang et al. 2009). This genus has scales present on the appendages according to *S. bicolor* (Zhang et al. 2009), however, Jin et al. (2017) proposed “scales present or absent on appendage” based on *Sinhomidia guangxiensis* Jin et al., 2017, which lacked such scales.

The genera *Sinhomidia* and *Homidia* Börner, 1906 are closely related, the scaled *Sinhomidia* being recognised as sister group of unscaled *Homidia* (Zhang et al. 2014a). This close relationship is supported by morphological, molecular and ecological evidence. Firstly, these two genera share several morphological characters, such as cephalic chaetotaxy on the dorsal side, eyebrow-like macrochaetae on abdominal segment IV, dens with inner spines in adults, smooth labial chaetae e and l_1_, the subapical mucronal tooth larger than the apical one, a bilobed bulb on antennal apex (Zhang et al. 2009, Zhang et al. 2014b), bothriotricha formula (2/3/2 on abdominal segment II/III/IV), and sensory chaetotaxic formula on tergal segments (3, 2/2, 2, 3, ? (sensory chaetae on abdominal segment IV variable), 3). They are easily misidentified as *Homidia* in the field in view of their colour pattern and body form. Secondly, recent molecular phylogenetic analyses revealed a close relationship between them based on mitochondrial, ribosomal and nuclear gene fragments (Zhang et al. 2014a, 2015, 2016). Also, Ding et al. (2019) placed *Sinhomidia* within the clade of *Homidia* based on mitochondrial COI, 16S rRNA and nuclear 28S rRNA D1-2. Thirdly, these two genera live in similar habitat, and are usually found in leaf litter in tropical to subtropical bioclimates. *Sinhomidia* with two known species and the new species described here is much less diversified than *Homidia* which has about 37 species in China (Zhang et al. 2009, Jin et al. 2017, Ma and Pan 2017). *Sinhomidia* can be easily discriminated morphologically from *Homidia* by scales on the terga and slightly modified accessory microchaetae of the bothriotrichal complex (Zhang et al. 2009).

The genus *Sinhomidia* is endemic to China. The type species (*S. bicolor*) was first recorded from Taiwan (Yosii 1965), and subsequently a female specimen was found in Anhui Province (Zhang et al. 2009) and male specimens in Guangxi Province (Jin et al. 2017). To date, only two species of this genus have been described (*S. bicolor* and *S. guangxiensis*), and the chaetotaxy of larvae has not been revealed. Here, a third species from Guangdong Province is described, together with its first instar larva. We provide a detailed comparison between the new species and the two known species, and we compare the first instar chaetotaxy among nine species of family Entomobryidae. In addition, a key to the recorded species of *Sinhomidia* is provided.

## Materials and methods

Specimens were sieved from leaf litter onto a tray in the field, collected by an aspirator, and stored in 99% ethanol at -20 °C. Specimens were photographed using a Nikon DS-Fi1 camera mounted onto a Nikon SMZ1000 stereomicroscope, then cleared in lactic acid, mounted in Hoyer’s medium under a coverslip, and examined under phase contrast using a Nikon 80i microscope. Lengths were measured from specimens on slide by NIS-Elements Documentation 3.1 software. Photos, illustrations and labels were enhanced by photoshop CS5 (Abode Systems).

Dorsal chaetotaxy is provided for only one side of the body. The nomenclature of cephalic chaetotaxy follows Szeptycki’s system (Szeptycki 1973), labial palp follows Fjellberg (1998), labial chaetae follow Gisin (1967), and dorsal thoracic and abdominal chaetotaxy follows Szeptycki (1979).

Abbreviations:

**Abd.** abdominal segment;

**Ant.** antennal segment;

**Gr.** group;

**mac** macrochaeta(e);

**mic** microchaeta(e);

**ms** specialised microchaeta(e);

**sens** specialised ordinary chaeta(e);

**Th.** thoracic segment;

**VT** ventral tube;

## Taxonomy

### Sinhomidia uniseta sp. nov.

Taxon classificationAnimaliaCollembolaEntomobryidae

AB252C17C19155FD842F0E5CCA3D144D

http://zoobank.org/656D27C0-C669-4E19-9399-9CDBA73CF9E5

Figs 1–9 , 10–20 , 21–24 , 25–32 , 33–42 , 43–45

#### Description

**Figures 1–9.**
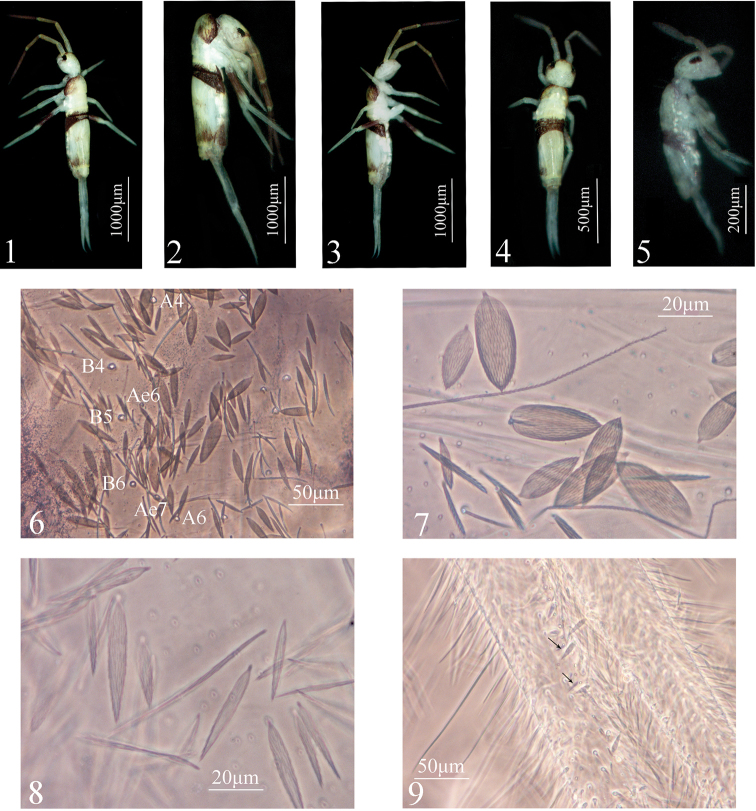
*Sinhomidia uniseta* sp. nov. **1–5** Habitus: **1** dorsal view of adult **2** lateral view of adult **3** ventral view of adult **4** dorsal view of subadult **5** lateral view of 1^st^ instar larva **6** postero-median scales on Abd. IV **7** scales and bothriotrichal complex on Abd. III **8** scales on manubrium **9** dental spines.

**Figures 10–20.**
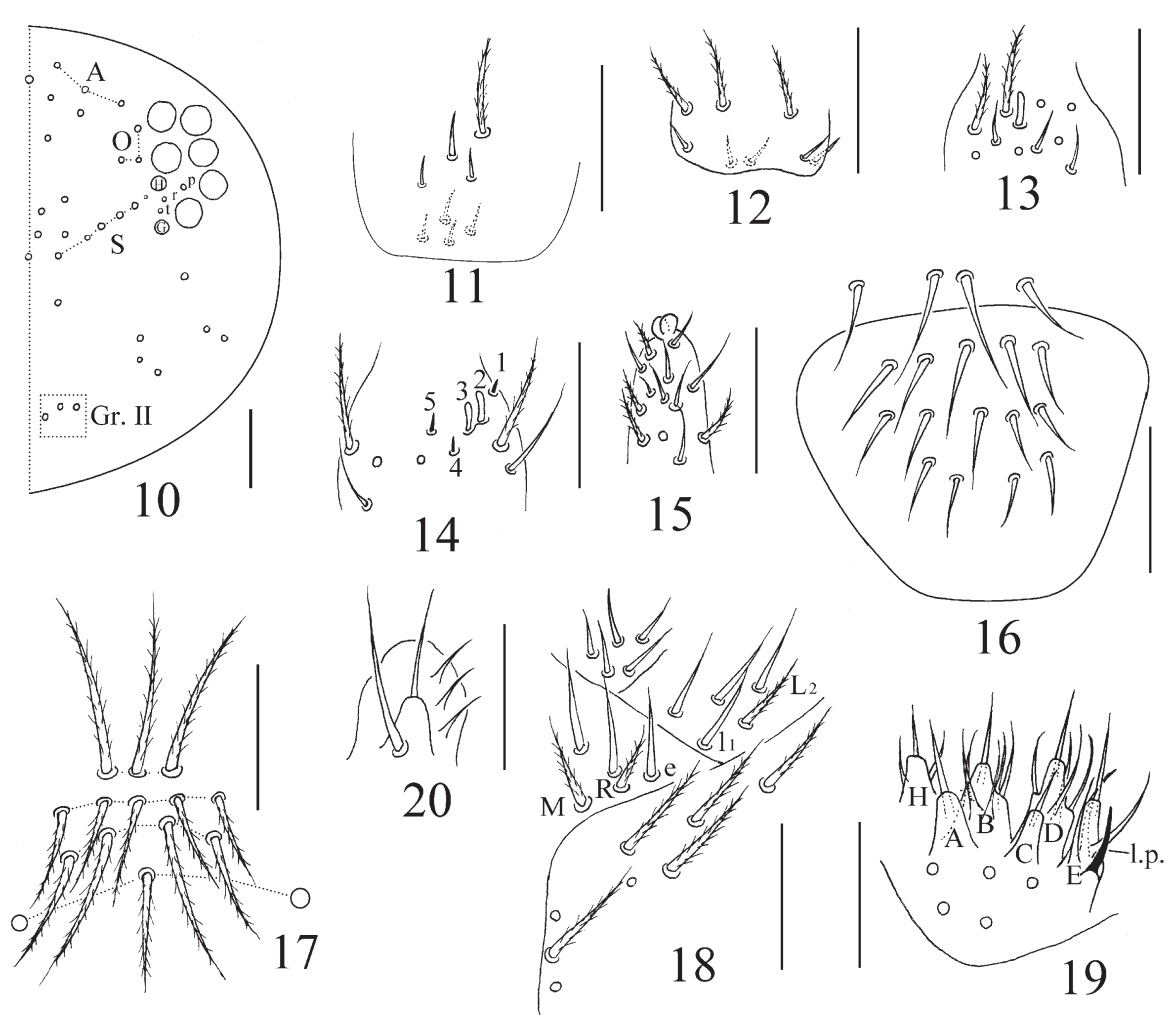
*Sinhomidia uniseta* sp. nov. **10** Cephalic chaetotaxy on dorsal side **11** basis of Ant. I **12** basis of Ant. II **13** distal part of Ant. II **14**Ant. III organ **15** distal part of Ant. IV **16** labrum **17** clypeal chaetotaxy **18** labium **19** labial palp **20** maxillary outer lobe **10–16** dorsal view **17–19** ventral view **20** lateral view. Scale bars: 50 μm.

**Figures 21–24.**
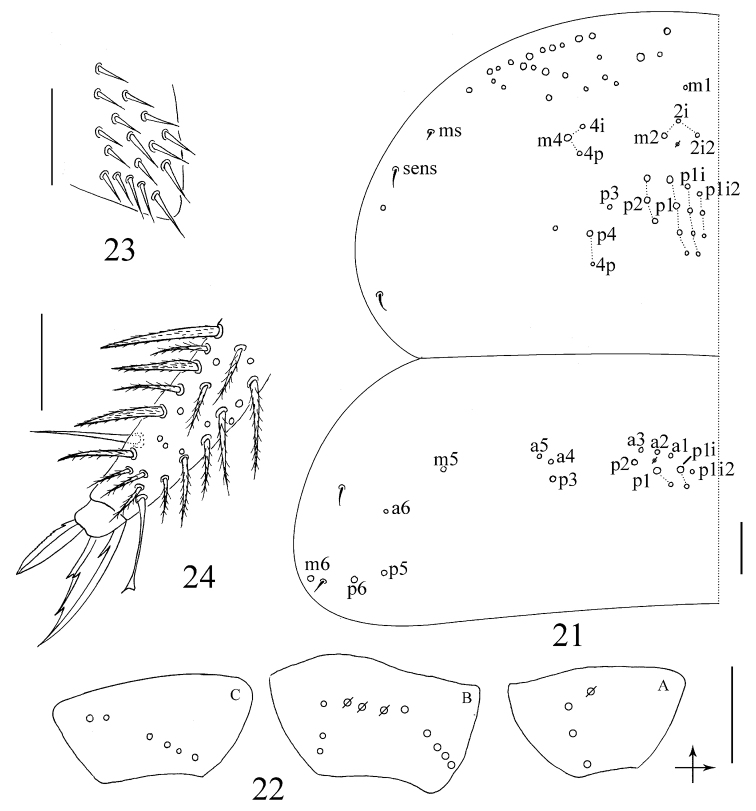
*Sinhomidia uniseta* sp. nov. **21** Chaetotaxy of Th. II–III tergites **22** Coxae (**A** fore leg **B** middle leg **C** hind leg) **23** Trochanteral organ **24** Distal part of tibiotarsus and claw of hind leg **21, 24** dorsal view **22, 23** lateral view. Scale bars: 50 μm.

**Figures 25–32.**
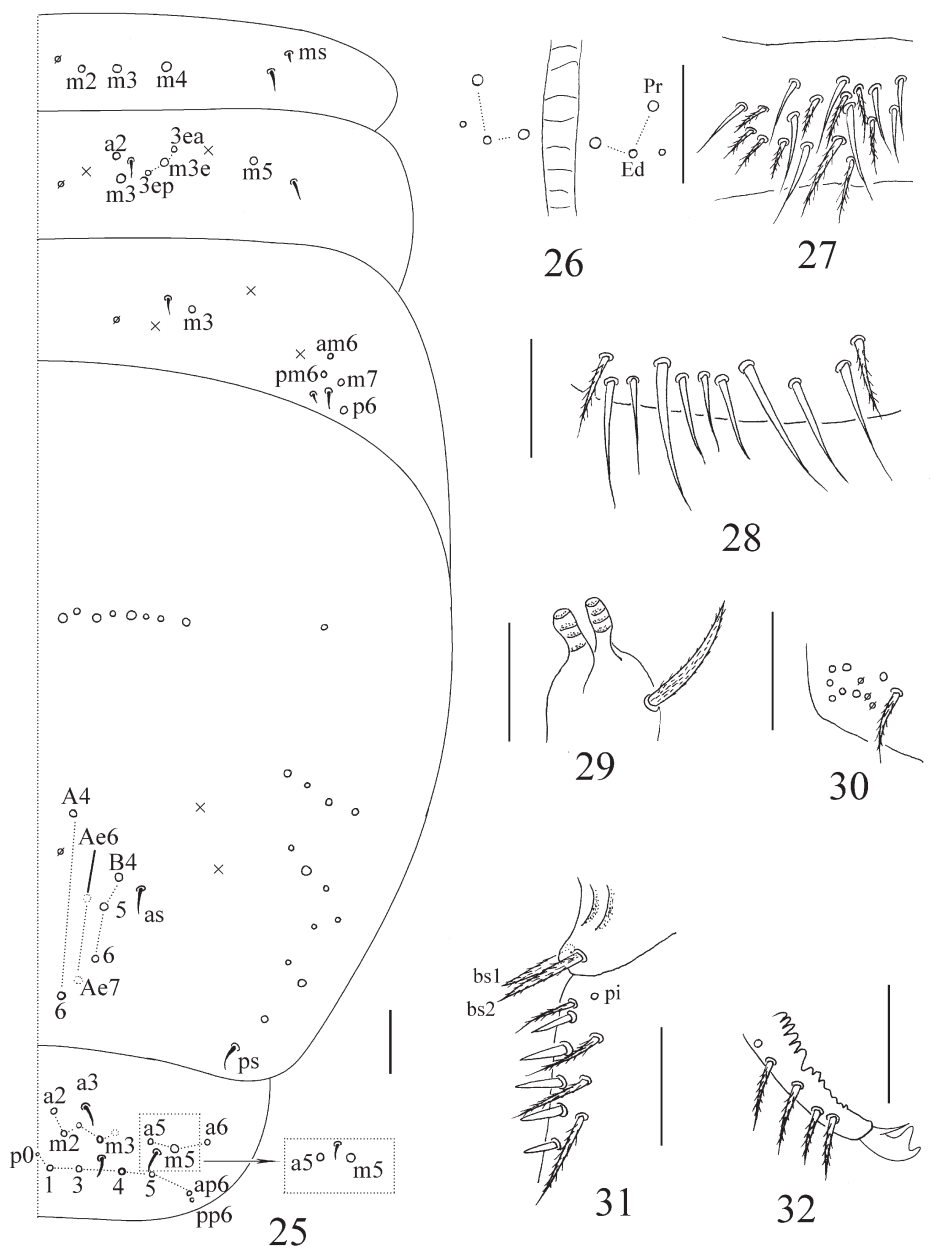
*Sinhomidia uniseta* sp. nov. **25** Dorsal chaetotaxy of Abd. I–Abd. V tergites **26** anterior face of ventral tube **27** lateral flap of ventral tube **28** posterior face of ventral tube **29** tenaculum **30** manubrial plaque **31** basal part of dens **32** distal part of dens and mucro **25, 26, 31, 32** dorsal view **28–30** ventral view **27** lateral view. Scale bars: 50 μm.

(adult and subadult). ***Size.*** Body length up to 2.51 mm. ***Scales.*** Scales pointed and coarsely striated, present on dorsal side of head, thorax and abdomen (Figs 6, 7), with fewer scales present on antennae and legs. Ventral side of manubrium with longer and narrower scales than those of dorsal side of body (Fig. 8). ***Colour pattern.*** Ground colour whitish in ethanol. Eye patches dark blue. Antennae with dark pigmentation, gradually darker from Ant. I to Ant. IV. A dark band between basal antennae. Lateral and anterior margin of Th. II, posterior half of Abd. II, whole Abd. III, posterior Abd. IV and femurs of the hind leg pigmented (Figs 1–3). Ventral side of body and VT without pigment (Fig. 3). Subadults with the same colour pattern as adults, but Th. II laterally and Abd. IV posteriorly lighter (Fig. 4). ***Head.*** Eyes 8+8, G and H smaller than others and always difficult to observe using a light microscope; three chaetae (p, r, and t) within eye patches, with p largest (Fig. 10). Antenna 2.10–2.45 times as long as cephalic diagonal; antennal segments ratio as I:II:III:IV = 1:1.48–1.91: 1.28–1.56:2.51–3.34. Ant. I basally with 3 dorsal and 4 ventral smooth mic (Fig. 11). Ant. II with 5 basal smooth mic (Fig. 12), and 1 longer (rarely 3) and 1 shorter rod-like distal S-chaetae (Fig. 13). Ant. III organ with 2 rod-like and 3 short guard S-chaetae (Fig. 14). Bulb on apical Ant. IV bilobed (Fig. 15). Prelabral and labral chaetae as 4/5, 5, 4, all smooth; labral papillae absent (Fig. 16). Clypeus with 15 mac in four lines, arranged as 3, 5, 4, 3 (Fig. 17). Cephalic chaetotaxy on dorsal side with 3 antennal (A), 3 ocellar (O) and 5 sutural (S) mac, Gr. II with 3 mac (Fig. 10). Chaetae on labium basis as MRel_1_L_2_, with e and l_1_ smooth; postlabial chaetae not expanded (Fig. 18). Five papillae A–E on labial palp with 0, 5, 0, 4, 3 guard chaetae, respectively; lateral process (l.p.) normal, with tip reaching apex of papilla E; hypostoma with 2 guard chaetae; proximal chaetae 5 (Fig. 19). Maxillary outer lobe with 1 apical chaeta, 1 subapical chaeta and 3 sublobal hairs on sublobal plate, subapical chaeta slightly larger than apical one (Fig. 20). ***Thorax.*** Complete body sens as 2, 2/1, 2, 2, 33, 3, ms as 1, 0/1, 0, 1, 0, 0. Th. II with 4 medio-medial (m1, m2, m2i and m2i2), 3 medio-sublateral (m4, m4i and m4p) mac and 3 S-chaetae (ms antero-internal to sens); posterior with 17–19 mac; p6 as mic. Th. III with 17–19 mac and 2 sens; p5, p6 and m6 as mac, p4 as mic (Fig. 21). Coxal macrochaetal formula as 3 (1 pseudopore)/4+1, 3 (3 pseudopores)/ 4+2 (pseudopore(s) unclear) mac (Fig. 22). Trochanteral organ with 24–34 smooth chaetae, 5–6 in ventral and 3–4 in posterior line (Fig. 23). Inner tibiotarsus with slightly ciliated chaetae. Tenent hairs clavate, slightly shorter than inner edge of unguis in length. Unguis with 3 inner and 2 lateral teeth, tooth on outer edge unclear. Unguiculus lanceolate with outer edge slightly serrate and the most basal tooth larger (Fig. 24). ***Abdomen.***Abd. IV 11–15 times longer than Abd. III along the dorsal axis. Abd. I with 3 mac (m2–4) and 2 S-chaetae (ms antero-external to sens). Abd. II with 5 central (a2, m3, m3e, m3ea and m3ep) and 1 lateral (m5) mac. Abd. III with 1 central (m3) and 4 lateral (am6, pm6, p6 and m7) mac, 2 sens and 1 ms. Abd. IV with 31 elongated and 2 normal length sens, and 10–14 mac arranged in anterior eyebrow-like line; postero-central area with 5 (7) mac (A4, A6, B4–6; Ae6 and Ae7 sometimes present). Abd. V with 3 sens, the middle one posterior to m3, the lateral one between chaetae a5 and m5 but shifted from anterior to posterior of a5 among the examined specimens; a1, a3, m3, m5, a5, m5, and a6 as mac, m3a sometimes as mac (Fig. 25). Anterior face of VT with many ciliated chaetae, 3+3 of them as mac, in a line connecting proximal (Pr) and external-distal (Ed) mac obliquely to median furrow (Fig. 26); lateral flap with 6–9 smooth and 10–11 ciliated chaetae on each side (Fig. 27); apical smooth chaetae on posterior face variable, five of examined specimens have 5 (2+1+2), one has 7, and another one has 9 (Fig. 28). Tenaculum with 4+4 teeth and 1 large, multi-laterally basally ciliated chaeta (Fig. 29). Manubrial plaque with 3 pseudopores and 8–10 ciliated chaetae (Fig. 30). Dens with 31–39 inner spines, basal chaetae (bs) spiny and multi-laterally ciliate, bs1 shorter than bs2, the morphology of chaeta pi unclear (Figs 9, 31). Mucro bidentate with subapical tooth larger than apical one; basal spine short, with tip reaching subapical tooth; distal smooth part of dens slightly shorter than mucro (Fig. 32).

#### Description of the first instar larva.

**Figures 33–42.**
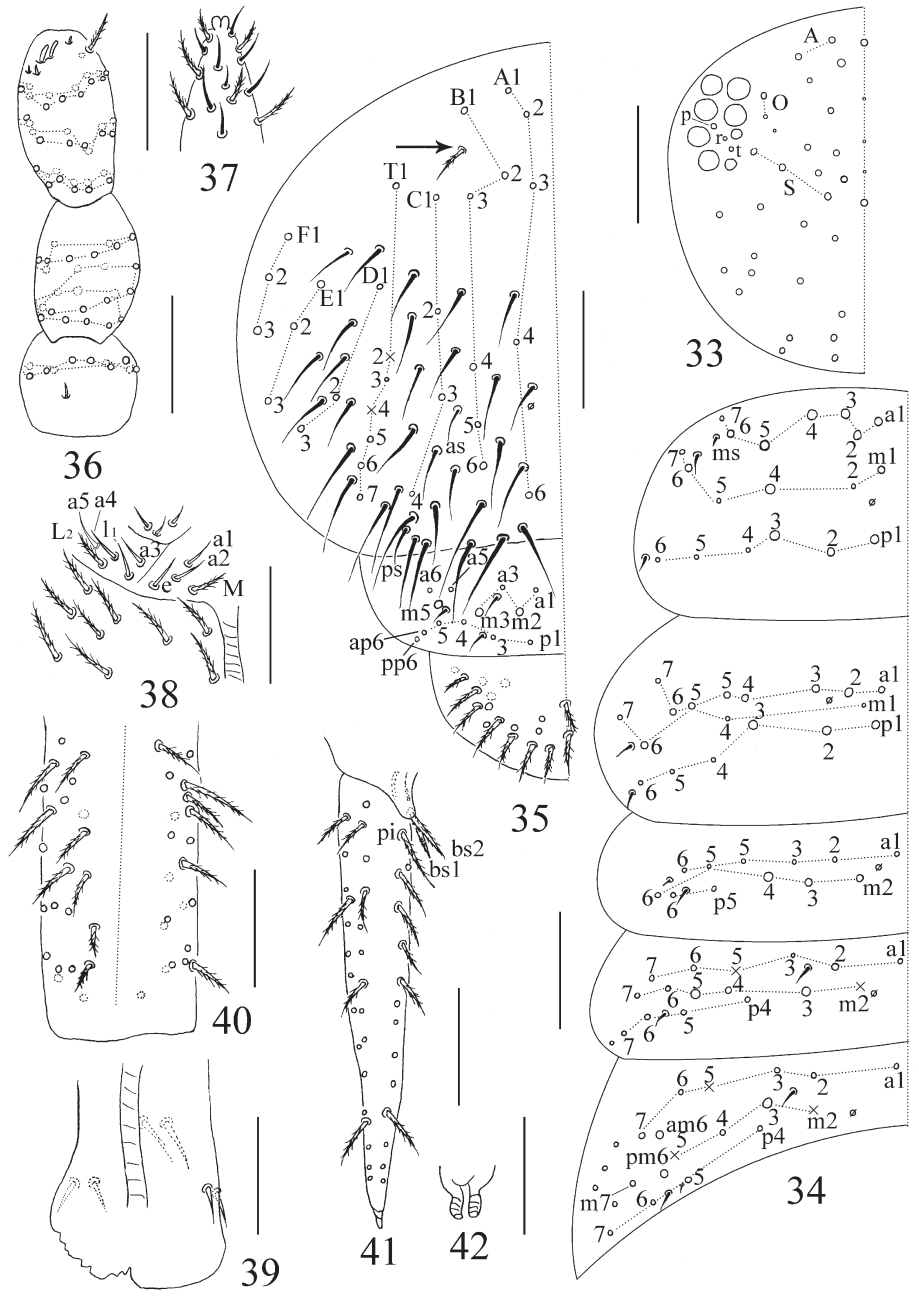
The first instar larvae of *Sinhomidia uniseta* sp. nov. **33** Dorsal cephalic chaetotaxy **34** chaetotaxy of Th. II–Abd. III tergites **35** chaetotaxy of Abd. IV–VI tergites **36** chaetotaxy of Ant. I–III **37** distal part of Ant. IV **38** labium **39** ventral tube **40** manubrium **41** dens **42** tenaculum **33–37, 39–42** dorsal view **38** ventral view. Scale bars: 50 μm.

**Figures 43–45.**
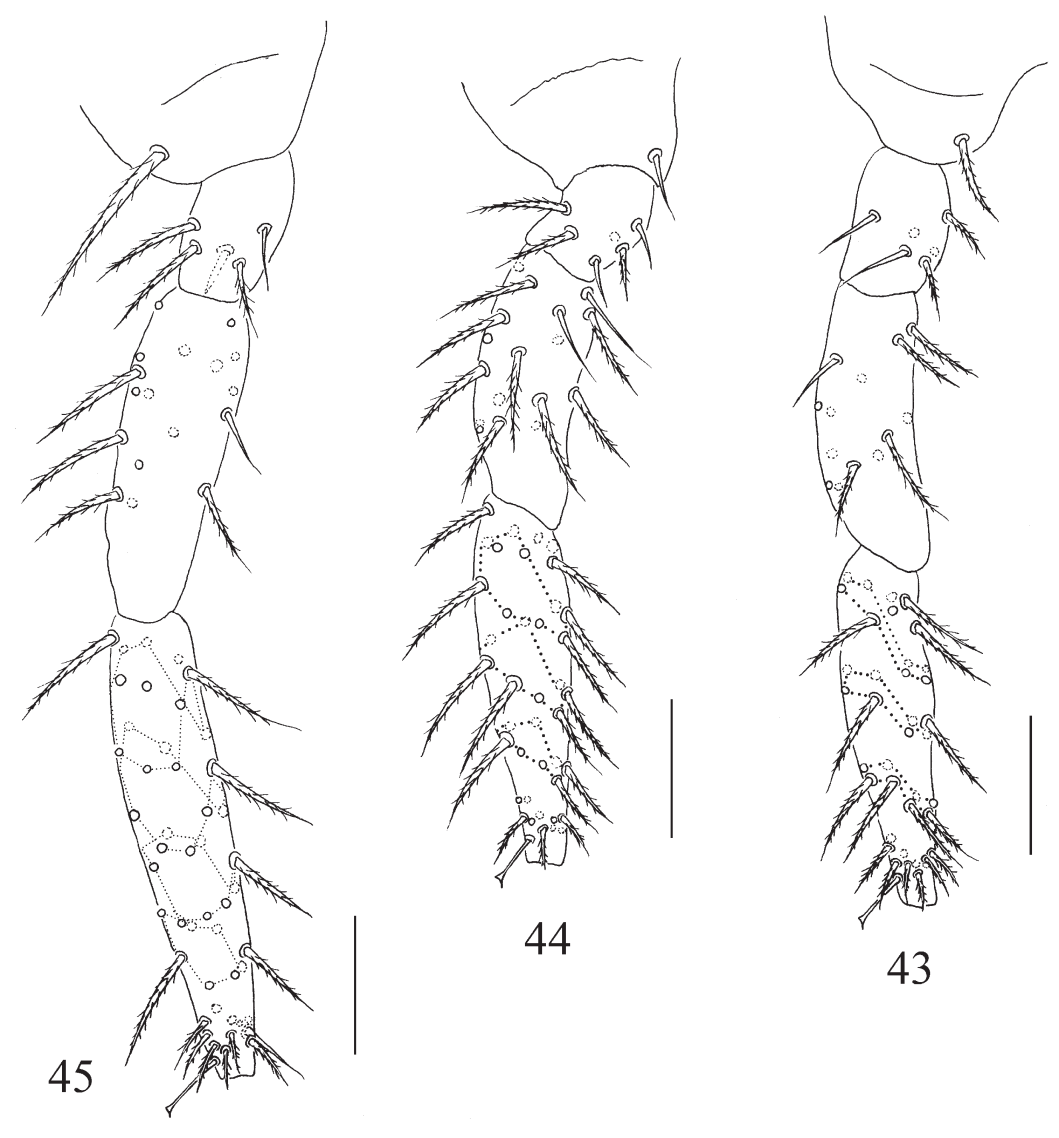
Left legs of the first instar of *Sinhomidia uniseta* sp. nov. **43–45** Anterior view of fore leg (**43**), mid leg (**44**) and hind leg (**45**). Scale bars: 50 μm.

***Size.*** Body length up to 0.79 mm. ***Colour pattern.*** Ground colour whitish, eye patches dark, antennae and femurs of hind legs with weak pigments, posterior half of Abd. II and whole Abd. III pigmented. The colour pattern is similar to that of adults, but paler overall (Fig. 5). ***Body.*** Body without scales. Complete tergal sens as 2, 2/1, 2, 2, 33, 3, ms as 1, 0/1, 0, 1, 0, 0. Cephalic chaetotaxy on dorsal side with 2 antennal (A), 2 ocellar (O) and 3 sutural (S) mac; eyes 8+8, eye patches with 3 chaetae (p, r, and t; p largest) (Fig. 33). Labium with 3 proximal chaetae, 4 chaetae (M, e, a_1_ and a_2_) in basomedial field and 5 chaetae (a3–a5, l_1_ and L_2_) in basolateral field, chaetae M and L_2_ ciliate, and others smooth; posterior area of labium with 9 ciliated mac (Fig. 38). Th. II with 7 anterior (a1–7), 6 median (m1–2, m4–7), and 6 posterior (p1–6) primary chaetae arranged in 3 rows; chaetae a7, m2, m5, m7 and p4–6 as mic, others as mac, and with 3 S-chaetae (ms antero-internal to sens). Th. III with 7 anterior (a1–7), 5 median (m1, m4–7), and 6 posterior (p1–6) primary chaetae arranged in 3 rows, and 2 S-chaetae; chaetae a7, m1, m4, m7, and p4–6 as mic, others as mac. Abd. I with 5 anterior (a1–3, a5–6), 5 median (m2–6), and 2 posterior (p5–6) primary chaetae arranged in 3 rows and 2 S-chaetae (ms antero-external to sens); chaetae m2–m4 as mac, others as mic. Abd. II with 6 anterior (a1–3, a5–7), 6 median (m2–7), and 4 posterior(p4–7) primary chaetae arranged in 3 rows, an additional chaeta external to p7 and 2 S-chaetae; chaetae a2, m3 and m5 as mac, a5 and m2 as bothriotricha, others as mic. Abd. III with 6 anterior (a1–3, a5–7), 7 median (m2–5, am6, pm6, m7), and 4 posterior (p4–7) primary chaetae arranged in 3 rows, 4 additional chaetae in lateral region, and 3 S-chaetae (1 ms and 2 sens); chaeta m3, am6 and pm6 as mac, m2, a5 and m5 as bothriotricha, others as mic (Fig. 34). Abd. IV with 5 (A1–4, A6), 6 (B1–6), 4 (C1–4), 7 (T1–7), 3 (D1–3), 3 (E1–3), and 3 (F1–3) primary ciliated chaetae arranged in 7 longitudinal lines, an additional ciliated chaeta between B1 and B2 (shown by arrow in Fig. 35), and 31 elongated and 2 normal sens; T2 and T4 as bothriotricha. Abd. V with 13 primary chaetae (m2, m3 and m5 as mac; a1, a3, a5, a6, p1, p3, p4, p5, ap6 and pp6 as mic) and 3 sens, the median sens posterior to m3. Abd. VI with 16 ciliated chaetae on one side and 2 along the median axis (Fig. 35). ***Appendages.***Ant. I with 11 ciliated chaetae arranged in one ring and 1 basal smooth chaeta. Ant. II with 26 ciliated chaetae, arranged in 3 rings (8/8/9), basis without smooth spiny chaetae. Ant. III with 38 ciliated chaetae arranged in 4 rings (from basis to apex of Ant. III as 11/12/13/2) and 5 S-chaetae (Ant. III organ). Ant. IV with many S-chaetae (more than two types) and ciliated chaetae, apical bulb bilobed (Fig. 37). Ventral tube with 2 smooth chaetae on the posterior face and on each lateral flap anterior face without chaetae (Fig. 39). Manubrium with 42 ciliated chaetae, dens with numerous ciliated chaetae, without inner dental spines; chaetae bs2 longer than bs1, pi ciliated and similar in length to that of other ciliated chaetae; mucro with subapical tooth larger than apical one, basal spine absent (Fig. 41). Tenaculum with 4+4 teeth and without chaetae (Fig. 42). Four segments of fore, middle and hind leg with numerous chaetae, coxae with 1, 1, 1 chaetae, pseudopore unclear; trochanters with 6 (2 smooth), 6 (2 smooth), 5 (1 smooth and 1 spine like) chaetae; femurs with 13 (1 smooth), 17 (2 smooth), 17 (at least 1 smooth) ciliated chaetae; tibiotarsus with 39 (10/8/8/8/4 ciliated and 1 tenent hair), 41 (10/8/8/8/6 ciliated and 1 tenent hair), 48 (10/8/8/8/8/4, 1 tenent hair and 1 inner smooth chaetae) ciliated chaetae (Figs 43–45).

#### Ecology.

Found in leaf litter of *Calamus thysanolepis*.

#### Holotype.

1♀ on slide, **China**, Guangdong Province, Guangzhou city, Tianhe District, Longdong reservoir, 23°14.134'N, 113°23.94'E, altitude 127±5 m, sample number 4661, collected by Z-X PAN and S-S ZHANG, 24-III-2018. **Paratypes.** 4♀1♂adults, 1 subadult and 1 first instar larva on slides and 3 adults in ethanol, same data as holotype. All types were deposited at the School of Life Sciences, Taizhou University.

#### Etymology.

Specific epithet refers to the single chaeta M on labial basis (uni + seta).

#### Remarks.

*Sinhomidia uniseta* sp. nov. can be easily distinguished from the other two species of the genus by the dark pigment present on the lateral and anterior margins of Th. II, posterior margin of Abd. II and whole Abd. III, labial single M chaeta, three mac and ms external to sens on dorsal side of Abd. I, 5 (7) mac in postero-median area of Abd. IV, middle sens posterior to m3 on Abd. V, and three teeth on inner side of unguis. Detailed differences between the three species of *Sinhomidia* are listed in Table 1.

**Table 1.** Detailed differences between the three species of genus *Sinhomidia*.

Characters	*S. uniseta* sp. nov.	*S. bicolor*	*S. guangxiensis*
**Colour pattern**			
Lateral stripes on head	absent	present	absent
Pigment on Th. II	along lateral and anterior margins, rarely whole	along lateral and anterior margins	whole
Pigment on Abd. I	posterior margin	whole	whole
Middle dark band dorsally on Abd. IV	absent	present	present
Posterior dark band dorsally on Abd. IV	discontinuous	continuous	continuous
Scales on antennae, legs and manubrium	present	present	absent
Maximum body length (mm)	2.5	3.4	2.4
**Chaetotaxy**			
Chaeta M on labrum	undoubled	doubled	doubled
Chaetae a2 and a5 on Abd. I	absent	absent	present
Chaeta a3 on Abd. II	mic	mic	mac
Postero-medial mac on Abd. IV	5 (7)	9	8
Inner teeth of unguis	3	4	4
Smooth/ciliated chaetae on each lateral flap of ventral tube	5–9/10–11	14/14	3/13

## Discussion

### Close relationship revealed by the chaetotaxy of adults and first instar between *Sinhomidia* and *Homidia*

The genus *Sinhomidia* was named referring to many features shared with *Homidia* (Zhang et al. 2009). *Sinhomidia* is regarded as sister group of *Homidia* (Zhang et al. 2014a), and is considered to be scaled *Homidia* (Zhang et al. 2014b). The genera *Sinhomidia* and *Homidia* share many features, such as colour pattern; chaetotaxy of head, labium, labrum, terga; S-chaetotaxic pattern, bothriotrichal pattern; and morphology of Ant. IV bulb, claw and mucro of adults (Pan et al. 2011, Zhuo et al. 2018). Phylogeny supports this close similarity (Zhang et al. 2014a, b, 2016, Zhang and Deharveng 2015, Ding et al. 2019). Here, we show that chaetotaxy of the first instar larva is also more similar between *Sinhomidia* and *Homidia* than between *Sinhomidia* and the species of the other six genera within family Entomobryidae where it has been described, including the number, morphology and relative location of primary tergal chaetae (Tab. 2).

These two genera differ nevertheless by several characters, such as scales (present in *Sinhomidia* versus absent in *Homidia*) (Figs 6–8), number of guard chaetae on labial papilla E (3 in *Sinhomidia* versus 4 in *Homidia*) (Fig. 19), bothriotrichal complex (slightly modified accessory mic of *Sinhomidia* versus not modified in *Homidia*) (Fig. 7), the relative position of posterior mac (p series) of Th. II–III (close to m series in *Sinhomidia*, versus close to posterior margin in *Homidia*) (Fig. 21), the number of mac on the dorsal side of Abd. I (3–5 in *Sinhomidia* versus 9–11 in *Homidia*), and length ratio Abd. IV/Abd. III (11–15 in the new species, but less than 10 times in *Homidia*, generally) (Zhang et al. 2009, Pan et al. 2011, Pan and Shi 2015, Jin et al. 2017, Zhuo et al. 2018).

**Table 2.** Comparison of the first instar larvae among nine species of Entomobryidae.

Tergite	Chaetae	*S. un*	*H. q*	*H. j*	*S. um*	*S. b*	*O. f*	*H. n*	*E. m*	*P. a*
Th. II	m1	mac	mac	mac	mac	mac	mac	mic	mac	mac
	m2	mic	mic	mic	mic	mic	mic	mic	mic	scale
	p4	mic	mic	mic	mic	mic	mic	mic	mic	mic
	p5	mic	mac	mac	mic	mac	mac	mic	mic	mac
	P6	mic	mic	mic	mic	mic	mic	mic	mic	mic
Th. III	a1	mac	mac	mic	mic	mac	mac	mic	mic	mic
	a2	mac	mac	mac	mac	mac	mac	mic	mic	mic
	a3	mac	mac	mac	mac	mac	mic	mic	mic	mic
	a4	mac	mac	mac	mac	mac	mac	mic	mic	–
	a5	mac	mac	mac	mac	mac	mac	mac	mac	mac
	m1	mic	mic	mic	mic	–	mic	mic	–	–
	m2	–	–	–	–	mac	–	–	mic	mac
	m5	mac	mac	mic	mic	mic	mic	mic	mic	mic
	p1	mac	mac	mac	mac	mac	mac	mac	mic	mac
	p2	mac	mac	mac	mac	mac	mac	mac	mic	mac
Abd. I	a4	–	–	–	–	–	–	–	mic	–
	a5	mic	mic	mic	mic	mic	mic	mic	–	mic
	m2	mac	mac	mac	mic	mac	mac	mac	mic	mac
	m4	mac	mac	mac	mac	mac	mac	mic	mac	mac
	m6	mic	mic	mic	mic	mic	mic	mic	mic	mic
Abd. II	a1	mic	mic	mic	mic	mic	mic	mic	mic	mic
	a2	mic	mic	mic	mic	mac	mac	mic	mic	mac
	a6	mic	mic	mic	mic	mic	mic	mic	–	mic
	a7	mic	mic	mic	mic	mic	mic	mic	mic	–
	m3	mac	mac	mac	mac	mac	mac	mac	mac	mac
	m4	mic	mic	mic	mic	mic	–	–	mic	mic
	m5	mac	mac	mac	mac	mac	mac	mic	mic	mac
	m6	mic	mic	mic	mic	mic	mic	mic	mac	mic
	m7	mic	mic	mic	mic	–	mic	mic	–	mic
	p4	mic	mic	mic	mic	mic	mic	mic	–	–
	p5	mic	mic	mic	mic	mic	mic	–	–	–
Abd. III	a1	mic	mic	mic	mic	mic	mic	mic	mic	mic
	a2	mic	mic	mic	mic	mic	mic	mic	mic	mic
	a7	mic	mic	mic	mic	mic	mic	mic	–	mic
	m4	mic	mic	mic	mic	mic	–	mic	–	mic
	am6	mac	mac	mic	mic	mic	mac	mac	mac	mac
	pm6	mac	mac	mac	mic	mac	mac	mac	mac	mac
	p4	mic	mic	mic	mic	–	mic	mic	mic	–
	p5	mic	mic	mic	mic	mic	mic	–	mic	–
Abd. IV	A4	mac	mic	mic	–	–	–	–	mic	–
	A5	–	mic	–	mic	mic	–	–	–	mic
	A6	mac	mic	mac	mic	mic	–	mic	?	mic
	B4	mac	mac	mic	mac	mic	–	–	mic	mic
	B5	mac	mac	mac	mac	mac	mic	mic	mac	mac
	B6	mac	mic	mic	mic	mic	mic	–	mic	mic
	E2	mac	mac	–	–	mic	mac	mic	–	mic
	E3	mac	mac	mac	mac	mac	–	–	mac	mic
Abd. V	a1	mic	mic	mic	mic	mic	mic	mic	mic	mic
	a3	mic	mic	mic	mic	mic	–	–	–	–
	m2	mac	mac	mac	mac	mac	mac	mac	mic	mac
	m3	mac	mac	mac	mic	mac	mac	mac	mic	mac
	a5	mic	mic	mic	mic	mic	mac	mac	–	–
	m5	mac	mac	mac	mic	mac	–	mic	–	mac
	a6	mic	mic	mic	mic	mic	mic	mic	–	mic
	p1	mic	mic	mic	mic	mic	mic	mic	mic	mic
	p2	–	–	–	–	–	–	–	mic	–
	p3	mic	mic	mic	mic	mic	mic	mic	–	mic
	p4	mic	mic	mic	mic	mic	mic	–	mic	mic
	p5	mic	mic	mic	mic	mic	mic	–	–	–
	ap6	mic	mic	mic	mic	mic	mic	mac	mic	mic

Notes: *S. un*: *Sinhomidia uniseta* sp. nov.; *H. q*: *Homidia quadriseta* Pan, 2018; *H. j*: *Homidia jordanai* Pan et al., 2011; *S. um*: *Sinella umesaoi* Yosii, 1940; *S. b*: *Seira barnadi* (Womersley, 1934); *O. f*: *Orchesella flavescens* (Bourlet, 1839); *H. n*: *Heteromurus nitidus* (Templeton, 1836); *E. m*: *Entomobryoides myrmecophila* (Reuter, 1884); *P. a*: *Pseudosinella alba* (Packard, 1873); -: absent; ?: unclear (*H. q* refer to Zhuo et al. 2018; *H. j* refer to Pan et al. 2011; *S. um* and *S. b* refer to Zhang and Deharveng 2015; other four species refer to Szeptycki 1979).

### Are scales present on appendages of *Sinhomidia*?

Scales are intuitively considered to have evolved from ordinary chaetae, present in many species, and are important diagnostic characters for classification at the subfamilial and tribal levels of the family Entomobryidae. The tribe Willowsiini is well defined by the absence of dental scales (Zhang et al. 2009). However, the presence or absence of body scales for classification is not valid for Willowsiini (Zhang et al. 2014a). *Sinhomidia* is a member of Willowsiini by the absence of scales on dens, and differs from other genera by its dental spines. Two recorded species of *Sinhomidia* and the new species described here are consistent in the morphology of the scales and tip pointed and fusiform with coarse striations, but they do not agree well between them in whether the scales are present on appendages. They are present on appendages of *S. bicolor* and the new species, but absent on *S. guangxiensis* (Zhang et al. 2009, Jin et al. 2017). Referring to the examined specimens of the new species, a few scales are present on the basal segments of antennae and legs, and ventral side of manubrium; furthermore, scales on the manubrium are narrower and longer than on the dorsal side of the terga, and similar to normal chaetae (Fig. 8). Additionally, scales easily fall off after clearing, and their sockets are difficult to distinguish from those of normal chaetae when checked by light microscope. To confirm if *Sinhomidia* has scales present on appendages in all species, it would be necessary to check the holotype of *S. guangxiensis*.

### Key to the species of genus *Sinhomidia*

**Table:** 

1	Abd. I entirely dark pigmented, Abd. IV with a middle dark band, claw with 4 inner teeth, labial chaeta M doubled	**2**
–	Abd. I with posterior margin dark pigmented, Abd. IV without middle dark band, claw with 3 inner teeth, labial chaeta M undoubled	***S. uniseta* sp. nov.**
2	Head with lateral dark stripes, chaetae a2 and a5 absent on Abd. I	***S. bicolor***
–	Head without lateral dark stripes, chaetae a2 and a5 present on Abd. I	***S. guangxiensis***

## Supplementary Material

XML Treatment for Sinhomidia uniseta
